# Suppression of SREBP-1 Expression by Simvastatin Decreases Visfatin-Induced Chemoresistance to Sunitinib in Human Renal Carcinoma 786-O Cells

**DOI:** 10.3390/life12111890

**Published:** 2022-11-15

**Authors:** Te-Chuan Chen, Chen-Wei Huang, Chih-Yu Lo, Cheng-Nan Chen, Shun-Fu Chang, Yih-Yuan Chen

**Affiliations:** 1 School of Medicine, College of Medicine, National Sun Yat-sen University, Kaohsiung 804, Taiwan; 2 Division of Nephrology, Kaohsiung Chang Gung Memorial Hospital, Chang Gung University College of Medicine, Kaohsiung 833, Taiwan; 3 Department of Food Science, National Chiayi University, Chiayi 600, Taiwan; 4 Department of Biochemical Science and Technology, National Chiayi University, Chiayi 600, Taiwan; 5 Department of Medical Research and Development, Chang Gung Memorial Hospital Chiayi Branch, Chiayi 613, Taiwan

**Keywords:** renal cell carcinoma, sunitinib, SREBP-1, visfatin, simvastatin

## Abstract

The resistance of renal cell carcinoma (RCC) to sunitinib impedes the success of chemotherapy in cancer treatment. Although several sunitinib resistance mechanisms have been proposed, little is known concerning the impact of obesity and adipokines in RCC cells. The upregulation of sterol-regulatory element-binding protein-1 (SREBP-1) has been reported to modulate the progression of tumor cells. The present study investigated the effect of visfatin on sunitinib-induced cytotoxicity in RCC cells through SREBP-1 expression. We found that visfatin-induced Akt and p70S6K activation increased SREBP-1 expression in 786-O cells. The visfatin-induced SREBP-1 mRNA and protein levels were attenuated through the inactivation of Akt and p70S6K by pharmacological inhibitors. In addition, the SREBP-1 knockdown using siRNA enhanced the cytotoxic effects of sunitinib. Our results also revealed the roles of simvastatin in attenuating the effects of visfatin on 786-O cells by inhibiting the production of reactive oxygen species. In particular, simvastatin co-treatment increased the cell cytotoxicity of sunitinib in visfatin-treated 786-O cells, which were associated with down-regulation of SREBP-1 expression. Our results suggest an important role of SREBP-1 in visfatin-induced drug resistance of RCC cells to sunitinib. The cytotoxic mechanism of simvastatin on RCC cells may provide a new strategy to improve therapeutic outcomes for the RCC treatment.

## 1. Introduction

Renal cell carcinoma (RCC) is the most common malignant tumor of adult kidneys, responsible for about 85% of diagnoses [1]. Although numerous recommendations for prevention have been put forward in many countries, a large proportion of RCC patients remain in the advanced and metastatic stages with a very low survival rate [2]. Despite the progress RCC chemotherapy has made, the occurrence of drug resistance in cancer cells remains a difficult challenge for cancer treatment and mortality [3]. Hence, there is an urgent need for further investigations on early diagnosis markers and chemotherapy resistance mechanisms to improve clinical treatment strategies and practices for RCC patients. Sunitinib is the most commonly used first-line therapy for patients with metastatic RCC. While there is not much significant improvement in survival rates of RCC patients, 30% of patients developed resistance to sunitinib; the other 70% of patients who responded initially also developed resistance within 6–15 months [4]. The effects involved in sunitinib resistance remain unclear. Therefore, it is necessary to elucidate the underlying mechanisms of sunitinib resistance of RCC cells.

Visfatin is a recently discovered adipocytokine, mainly produced and secreted by visceral adipose tissue. It can induce insulin-like effects that bind to and activate insulin receptors in vivo and in vitro [5]. The visfatin gene can be upregulated in adipocytes and is mainly expressed in the visceral adipose tissue [6]. With their abundance and proximity to tumor cells, adipocytes affect the behavior of cancer cells, including tumor progression and metastasis [7]. In clear cell RCC, visfatin is up-regulated in cancer tissues than in adjacent normal tissues [5]. Patients with thyroid malignancies also have higher levels of visfatin expression, in which visfatin appears to be associated with advanced tumors [7]. In addition, the expression of visfatin is increased in experimental myeloma bones compared to non-myeloma bones [8]. Several epidemiological studies have linked the occurrence of kidney cancer with obesity [6]. Although there is a specific link between obesity and RCC, the mechanism by which obesity increases the risks of RCC remains unclear.

Sterol regulatory element-binding proteins (SREBPs) are key transcription factors that induce lipid-producing gene expression responsible for lipid and energy metabolism [9]. SREBP-1 has been known to promote tumor growth and lipid biosynthesis in several types of cancers [10]. It has been reported that clear cell RCC shows elevated adipogenesis in cancer tissues [11]. However, it is not yet fully understood how lipid synthesis is associated with RCC progression. While SREBP-1 upregulation promotes adipogenesis and cell proliferation, pharmacological inhibition of SREBP-1 attenuates these effects in RCC cells [12]. In addition, the expression of SREBP-1 mRNA and adipogenic genes were both increased in RCC patients, accompanied by tumor progression and poor prognosis [13]. Since SREBP plays a crucial role in RCC tumorigenesis, inhibiting SREBPs may become useful in treating RCC.

Statins, mainly used to reduce plasma cholesterol levels and ameliorate cardiovascular function have been repurposed for the prevention and treatment of tumors. Simvastatin has been shown to improve the prognosis and increase cancer survival rates [14]. The antitumor effect of simvastatin has been investigated in monotherapy or in combination with chemotherapeutic agents [15]. Our present study aimed to investigate the regulatory role of visfatin in the RCC cell cytotoxicity of sunitinib. It was shown that visfatin stimulation activates Akt and p70S6K to upregulate SREBP-1 through reactive oxygen species (ROS) in RCC 786-O cells. This SREBP-1 induction could further decrease the sensitivity of 786-O cells to sunitinib. Furthermore, by inhibiting the production of ROS, simvastatin effectively reverses the visfatin-induced drug resistance of RCC cells. Our findings found that SREBP-1 upregulation has an important role in the development of the drug resistance of RCC cells to chemotherapeutic agents.

## 2. Materials and Methods

### 2.1. Materials

Cell culture media, FBS, antibiotics, and all other cell culture materials were purchased from Gibco (Grand Island, NY, USA). Antibodies against phospho-Akt (#4051; 1:1000 dilution), Akt (#2920; 1:2000 dilution), phospho-p70S6K (#9204; 1:1000 dilution), p70S6K (#9202; 1:1000 dilution), and β-actin (#8457; 1:3000 dilution) were purchased from Cell Signaling Technology (Beverly, MA, USA). Antibody against SREBP-1 (sc-13551, 1:1000 dilution), the non-targeting siRNA (sc-37007) and siRNA for SREBP-1 mRNA (sc-36557) were purchased from Santa Cruz (Dallas, TX, USA). Protein kinase inhibitors (LY294002 for Akt and rapamycin for p70S6K), as well as all other reagents of analytical grade used in this study, were obtained from Sigma (St. Louis, MO, USA).

### 2.2. Cell Culture

The human RCC cell line 786-O cells were purchased from the cell bank of the Taiwan Food Industry Research and Development Institute (Hsinchu, Taiwan). The 786-O cells were maintained in Dulbecco’s Modified Eagle Medium containing 10% FBS with penicillin/streptomycin at 37 °C in a 5% CO_2_ atmosphere.

### 2.3. MTT Assay

Cytotoxicity evaluation and cell growth inhibition was assessed with MTT (3-(4,5-dimethylthiazol-2-yl)-2,5-diphenyltetrazolium bromide) assay. The 786-O cells were adjusted to 1 × 10^5^ cells/mL and were plated in 96-well microplates. After treatment, the MTT solution (0.5 mg/mL) in serum-free medium was added to the wells and further incubated for 4 h at 37 °C. The purple formazan crystals were dissolved in DMSO, and the absorbance of the wells was measured immediately at 570 nm using a spectrophotometer [16].

### 2.4. Real-Time Quantitative PCR

Total RNA was isolated by the Trizol reagent. cDNA was synthesized from 1 μg of total RNA by using a high-capacity reverse-transcription kit after the RNA purity and integrity were checked. A real-time PCR assay of the indicated genes was carried out by using the SYBR Green Master Mix (Thermo, Waltham, MA, USA) in 96-well plates. The following oligonucleotide primers of the indicated genes were: SREBP-1 (positive: 5′-CAGGT ACCGA GTTCT GGTGT GTTGG GCCA-3′; negative: 5′-ACTGC TAGCC GCGCT GCCGC CTCGC TAG-3′) and GAPDH (positive: 5′-AGGTG AAGGT CGGAG TCAAC-3′; negative: 5′-CCATG TAGTT GAGGT CAATG AAGG-3′). The GAPDH gene was employed as the internal control. All RT-PCR were determined in duplicate from three independent experiments. The relative expression of the target genes was calculated using ΔΔCt method and normalized to GAPDH mRNA levels [17].

### 2.5. Western Blot Analysis

The 786-O cells were seeded overnight at density of 1 × 10^6^ cells in 60-mm culture dishes before treatment. After treatment, cells were harvested and the total protein was extracted with ice-cold lysis buffer (Thermo Scientific, Rockford, IL, USA). Protein concentration was quantified according to the method of the Bradford protein assay kit (Bio-Rad, Hercules, CA, USA). Equal amounts of total protein lysates were loaded and separated by sodium dodecyl sulfate-polyacrylamide gel electrophoresis (SDS-PAGE) gel and blotted onto nitrocellulose paper. The immunoreactive bands of designated primary antibodies were probed with secondary antibodies and detected with an enhanced chemiluminescence (ECL) kit [18].

### 2.6. Dominant Negative (DN)-Akt and siRNA Transfection

For siRNA transfection, 786-O cells were cultured in DMEM supplemented with 10% FBS (without antibiotics) overnight and then transfected with the control siRNA or siRNA against SREBP-1 for at least 72 h using lipofectamine RNAiMAX (Invitrogen, Carlsbad, CA, USA) [15]. For DN-Akt transfection, 786-O cells were transfected with empty vector or DN-Akt plasmids. Transfections were carried out with Lipofectamine 2000 reagent (Invitrogen, Carlsbad, CA, USA) according to the manufacturer’s instructions. Cells were processed 48 h after transfection [19].

### 2.7. ROS Measurement

Cellular levels of ROS were detected in a 96-well plate by a fluorescent measurement system following the manufacturer’s instruction. After seeding 786-O cells (2 × 10^4^ cells/well) in the 96-well plate, the cells were washed with HBSS. Then, an H2DCFDA (5 mM) staining buffer was added, and the plate was incubated at 37 °C for 1 h in the dark. The fluorescence intensity was measured using a microplate reader with excitation and emission spectra of 485 and 535 nm, respectively [20].

### 2.8. Statistical Analysis

Results are presented as mean ± standard error (S.E). The data were analyzed for significance by one-way analysis of variance (ANOVA) followed by a Scheffe’s test for multiple comparisons. *p* values less than 0.05 were indicated as significant.

## 3. Results

### 3.1. Visfatin Increases the SREBP-1 Expression in 786-O Cells

**Figure 1:**
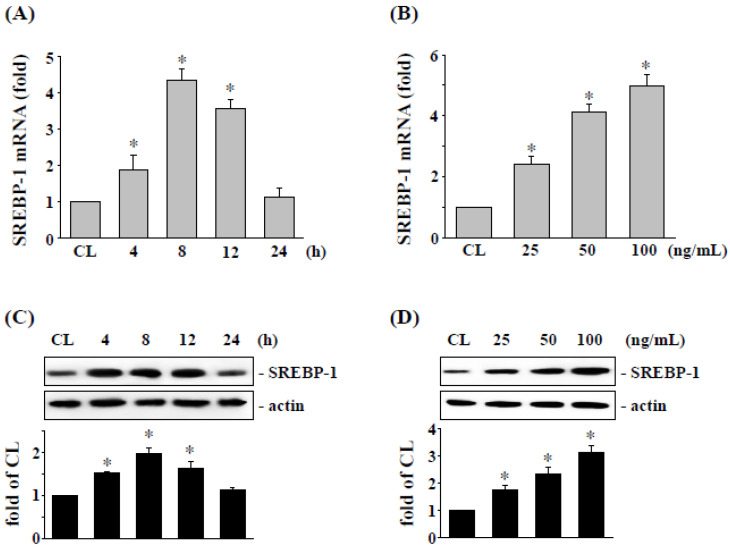
Visfatin induced SREBP-1 mRNA and protein expressions in a dose- and time-dependent manner in 786-O cells. Human RCC 786-O cells were kept as control (CL) or (**A**,**C**) treated with visfatin (50 ng/mL) for 0–24 h as indicated, or (**B**,**D**) treated with different doses of visfatin (0–100 ng/mL) for 8 h. The mRNA (**A**,**B**) and protein (**C**,**D**) expressions of SREBP-1 were examined by real-time PCR and Western blot analysis, respectively. Data in (**A**,**B**) were shown as mean ± SEM from three independent experiments. Results in (**C**,**D**) were representative of three independent experiments with similar results. * *p* < 0.05 was defined as statistically significant. The uncropped bolts are shown in Supplementary Materials.

To elucidate the possible effect of visfatin in cancer cells, the expression of SREBP-1 in RCC was analyzed in 786-O cells. RCC 786-O cells were stimulated with visfatin and cultured for 4–24 h. The expression of mRNA and protein of SREBP-1 were assessed by real-time PCR and Western blotting analysis, respectively. Compared to the untreated control cells, 786-O cells treated with visfatin remarkably enhanced expression of SREBP-1 mRNA (Figure 1A) and protein (Figure 1C) especially at 8 h. In addition, 786-O cells treated with different concentrations of visfatin for 8 h found that visfatin-induced SREBP-1 mRNA (Figure 1B) and protein (Figure 1D) were expressed in a dose-dependent manner.

### 3.2. SREBP-1 Expression Level Affects the Visfatin-Treated 786-O Cell Cytotoxicity of Sunitinib Treatment

**Figure 2:**
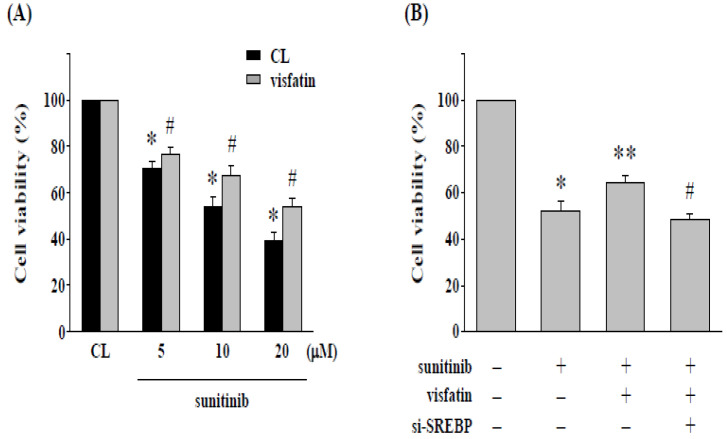
Gene knockdown of SREBP-1 in 786-O cells enhances the cytotoxicity induced by sunitinib. (**A**) Human RCC 786-O cells were pretreated with vehicle (PBS) or visfatin (50 ng/mL) for 1 h and then kept as control or treated with sunitinib (5–20 μM) for 24 h. * *p* < 0.05 was indicated as statistically significant vs. CL cells without sunitinib treatment. # *p* < 0.05 was indicated as statistically significant vs. sunitinib-treated cells without visfatin treatment. (**B**) Human RCC 786-O cells were transfected with control- or SREBP-1-specific siRNA (si-SREBP) for 48 h and then pretreated with vehicle (PBS) or visfatin (50 ng/mL) for 1 h and further kept as control or treated with sunitinib (10 μM) for 24 h. The viability of treated cells was examined by MTT assay. Data were shown as mean ± SEM from three independent experiments. * *p* < 0.05 was indicated as statistically significant vs. CL cells. ** *p* < 0.05 was indicated as statistically significant vs. sunitinib-treated cells. # *p* < 0.05 was indicated as statistically significant vs. cells co-treated with visfatin and sunitinib.

We assessed the effect of cytotoxicity of sunitinib on 786-O cells incubated with visfatin; the cells were cultured in a CL medium or stimulated by visfatin and treated with sunitinib (5-20 μM) for 24 h. The 786-O cells combined treatment with sunitinib and visfatin raised the cell viability of 786-O cells (Figure 2A). We further confirmed whether SREBP-1 expression affects the cell cytotoxicity induced by sunitinib combined with visfatin, 786-O cells were transfected with siRNA targeting SREBP-1 mRNA expression. Cells were treated with PBS (control cells) or visfatin (50 ng/mL) for 1 h followed by treatment of sunitinib (10 μM) for an additional 24 h. The cell viability was measured by MTT assay. Our results showed that the knockdown of SREBP-1 expression restores the cytotoxicity of sunitinib in 786-O cells attenuated by visfatin (Figure 2B).

### 3.3. Akt and p70S6K Signaling Regulates SREBP-1 Expression and Cell Cytotoxicity of 786-O Cells

**Figure 3:**
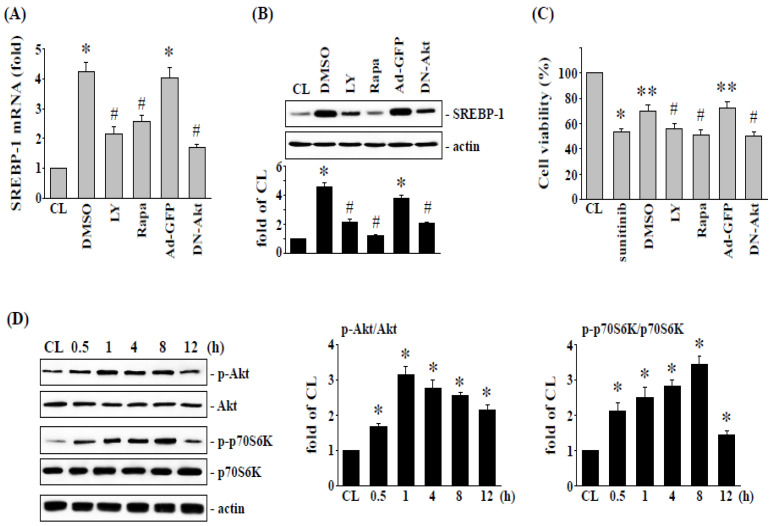
Akt and p70S6K signaling regulate visfatin-increased SREBP-1 expression and subsequent cell cytotoxicity of 786-O cells. Human RCC 786-O cells were pretreated with vehicle (DMSO) or inhibitors of Akt (LY294002, 25 μM) and p70S6K (rapamycin, 20 μM) for 1 h, or transfected with Ad-GFP or DN-Akt, and then treated with vehicle (PBS) or visfatin (50 ng/mL) for 1 h and further kept as control or treated with sunitinib (10 μM). The mRNA (**A**) and protein (**B**) expressions of SREBP-1 after 8 h treatment were examined by real-time PCR and Western blot analysis, respectively. * *p* < 0.05 was indicated as statistically significant vs. CL. # *p* < 0.05 was indicated as statistically significant vs. DMSO- or Ad-GFP-treated cells. (**C**) The viability of treated cells was examined by MTT assay after 24 h treatment. * *p* < 0.05 was indicated as statistically significant vs. CL cells. ** *p* < 0.05 was indicated as statistically significant vs. sunitinib-treated cells. # *p* < 0.05 was indicated as statistically significant vs. DMSO-treated cells. (**D**) RCC 786-O cells were kept as control (CL) or treated with visfatin (50 ng/mL) as times indicated, and then the phosphorylation of Akt and p70S6K was examined by Western blot analysis. Data in (**A**,**C**) were shown as mean ± SEM from three independent experiments. Results in (**B**,**D**) were representative of three independent experiments with similar results. The uncropped bolts are shown in Supplementary Materials.

We next examined whether the upregulation of SREBP-1 and the visfatin-induced reduction in cytotoxicity are regulated by PI3K/Akt and p70S6K signaling, visfatin-treated 786-O cells were treated with DMSO, specific inhibitors for Akt (LY294002), and p70S6K (rapamycin), or transfected with ad-GFP or DN-Akt, and treated with sunitinib (10 μM). 786-O cells pretreated kinase inhibitors caused ~80–90% reductions in the phosphorylation of the corresponding proteins. Pretreating 786-O cells with LY294002, rapamycin, or DN-Akt remarkedly decreased the visfatin-induced SREBP-1 mRNA (Figure 3A) and protein (Figure 3B) expression and improved the sunitinib-induced cell cytotoxicity (Figure 3C) compared with the vehicle controls (DMSO or Ad-GFP). Moreover, compared to control cells (CL), treatment of 786-O cells with visfatin (50 ng/mL) also induced a significant increase in Akt and p70S6K phosphorylation within 30 min and sustained activation for 8 h in human RCC 786-O cells (Figure 3D).

### 3.4. ROS Regulates SREBP-1 Expression and Cell Cytotoxicity of 786-O Cells

**Figure 4:**
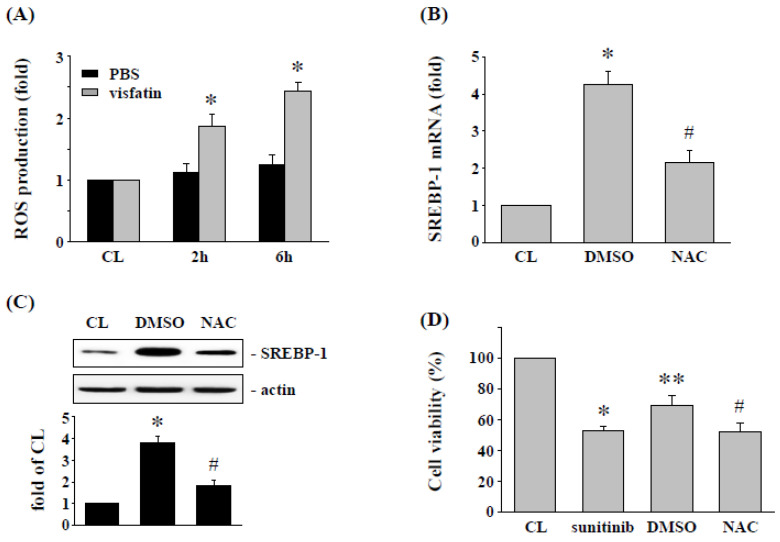
ROS regulates the SREBP-1 expression and subsequent sunitinib-induced death in visfatin-treated 786-O cells. (**A**) RCC 786-O cells were kept as control or treated with vehicle (PBS) or visfatin (50 ng/mL), and then the production of ROS was determined by ROS assay kit. * *p* < 0.05 was indicated as statistically significant vs. CL. (**B**,**C**) RCC 786-O cells were pretreated with DMSO or ROS inhibitor NAC (10 mM) for 2 h and then kept as control or treated with visfatin for 12 h, and then the expressions of (**B**) mRNA and (**C**) protein of SREBP-1 were examined by real-time PCR and Western blot, respectively. * *p* < 0.05 was indicated as statistically significant vs. CL. # *p* < 0.05 was indicated as statistically significant vs. DMSO-treated cells. (**D**) RCC 786-O cells were treated with DMSO or NAC (10 mM) for 2 h and then pretreated with vehicle (PBS) or visfatin (50 ng/mL) for 1 h and further kept as control or treated with sunitinib (10 μM) for 24 h. The viability of treated cells was examined by MTT assay. * *p* < 0.05 was indicated as statistically significant vs. CL cells. ** *p* < 0.05 was indicated as statistically significant vs. sunitinib-treated cells. # *p* < 0.05 was indicated as statistically significant vs. DMSO-treated cells. Data in (**A**,**B**,**D**) were shown as mean ± SEM from three independent experiments. Results in (**C**) were representative of three independent experiments with similar results. The uncropped bolts are shown in Supplementary Materials.

ROS has been shown to regulate the SREBP-1 expression and cell cytotoxicity of 786-O cells [21]. Hence, we determined whether ROS production modulates the effect of visfatin on SREBP-1 expression and further affects sunitinib-induced 786-O cell death. Intracellular ROS production was measured in 786-O cells after cells were treated with vehicle (PBS) or visfatin (50 ng/mL) for 8 h. It was found that visfatin stimulation of RCC 786-O cells increased ROS levels in a time-dependent manner compared with the untreated or PBS-treated control cells (Figure 4A). Cells were then pretreated with an ROS inhibitor (NAC, 10 mM) to inhibit ROS production. The 786-O cells were further treated with visfatin (50 ng/mL) for 8 h, and then the expression of SREBP-1 mRNA and protein was determined. The results showed that administration of NAC significantly inhibited the visfatin-induced expression of SREBP-1 mRNA (Figure 4B) and protein (Figure 4C). In addition, the NAC-treated cells were further co-stimulated with vehicle (PBS) or visfatin (50 ng/mL) for 1 h, followed by sunitinib (5 μM) for another 24 h. The viability of the treated 786-O cells was measured by MTT assay. Our results showed that NAC-treated cells restored the effects of visfatin on sunitinib-induced cytotoxicity in RCC 786-O cells.

### 3.5. Simvastatin Enhances Sunitinib-Induced Cell Cytotoxicity in Visfatin-Treated 786-O Cells

**Figure 5:**
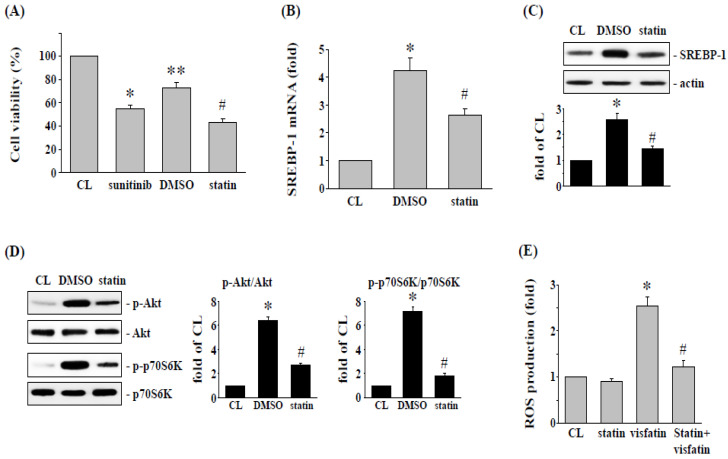
Simvastatin attenuates the visfatin effect on sunitinib-induced death in 786-O cells. (**A**) RCC 786-O cells were pretreated with vehicle (DMSO) or simvastatin (statin, 20 μM) for 1 h and then treated with vehicle (PBS) or visfatin (50 ng/mL) for 1 h and further kept as control or treated with sunitinib (10 μM) for 24 h. The viability of treated cells was examined by MTT assay. * *p* < 0.05 was indicated as statistically significant vs. CL cells. ** *p* < 0.05 was indicated as statistically significant vs. sunitinib-treated cells. # *p* < 0.05 was indicated as statistically significant vs. DMSO-treated cells. (**B**–**D**) RCC 786-O cells were pretreated with vehicle (DMSO) or simvastatin (statin, 20 μM) for 1 h and then treated with vehicle (PBS) or visfatin (50 ng/mL) for the times indicated (8 h for SREBP-1 expression and 6 h for ROS generation), and (**D**) 1 h for Akt and p70S6K phosphorylation. (**B**,**C**) The mRNA (real-time PCR) and protein (Western blot) expressions of SREBP-1, (**D**) Akt and p70S6K phosphorylation (Western blot), and (**E**) ROS production in human 786-O cells were determined. Data were shown as mean ± SEM from three independent experiments. Results in (**C**,**D**) were representative of three independent experiments with similar results. * *p* < 0.05 was indicated as statistically significant vs. CL. # *p* < 0.05 was indicated as statistically significant vs. DMSO-treated cells. The uncropped bolts are shown in Supplementary Materials.

It has been suggested that simvastatin may be an effective compound in cancer treatment [22]. Therefore, we speculated that the reduction of cytotoxicity of sunitinib in human RCC 786-O cells by visfatin might be affected by simvastatin. Cells were pretreated with vehicle (ethanol) or simvastatin (statin, 20 μM) for 1 h, followed by visfatin (50 ng/mL) for 1 h, and sunitinib (10 μM) for another 24 h. The results showed that the cytotoxicity of sunitinib, which was attenuated by visfatin stimulation in 786-O cells, was retrieved by simvastatin, and the visfatin-increased cell viability was restored (Figure 5A). Finally, RCC 786-O cells were pretreated with vehicle (ethanol) or simvastatin (20 μM) for 1 h, followed by visfatin (50 ng/mL) for various time points, the phosphorylation of Akt and p70S6K, mRNA and protein expression of SREBP-1, and generation of ROS were analyzed. It was found that treatment of 786-O cells with visfatin significantly increased the SREBP-1 mRNA and protein (Figure 5B,C) expression, activation of Akt and p70S6K (Figure 5D), and production of ROS (Figure 5E). However, these visfatin effects on human RCC 786-O cells were significantly suppressed by simvastatin.

## 4. Discussion

**Figure 6:**
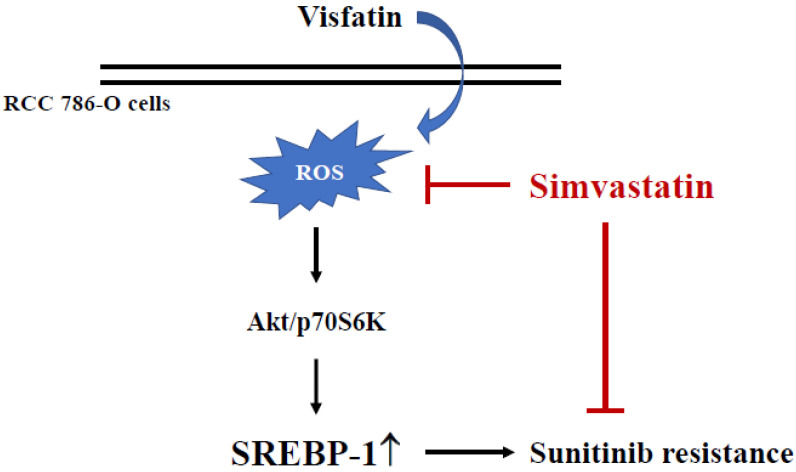
Schematic representation of a cytotoxic effect of simvastatin with sunitinib to RCC 786-O cells by suppressing visfatin-induced SREBP-1 upregulation.

The results of this study found that (i) SREBP-1 upregulation could be induced in 786-O cells amidst visfatin treatment, and this induction could consequently reduce sunitinib’s cytotoxicity to RCC 786-O cells. (ii) SREBP-1 expression and the subsequent decrease in cell death of visfatin induction were regulated by Akt and p70S6K phosphorylation. (iii) Simvastatin has a pivotal effect in controlling SREBP-1 expression in visfatin-treated 786-O cells by inhibiting ROS generation. This study elucidates a drug resistance mechanism in RCC cells that attenuates the sensitivity of RCC cells to sunitinib by upregulating the expression of SREBP-1, and that the resistance of RCC cells to sunitinib can be reversed by inhibiting the related mechanisms (Figure 6).

Evidence supports that the adipogenic phenotype is the main feature of cancer development [23]. Cancer cells are involved in the biosynthesis of lipids, which are produced from glucose and glutamine. The ability of cancer cells to participate in autonomous adipogenesis provides a major route through which cancer cells become independent of systemic regulation [23]. SREBP-1 is a transcription factor involved in the regulation of lipid homeostasis and the modulation of related gene expression [24]. Increased expression of SREBP-1 has been shown to be associated with tumorigenesis, metastasis, and the advanced stage progression in various types of cancers [25,26]. Previous studies revealed that SREBP-1 is overexpressed in the tumor tissues of RCC patients [10,27]. Further, SREBP-1 mRNA levels have also been reported to increase in a clean cell RCC [28]. In addition, SREBP-1 regulates cancer cell proliferation mediated by regulating intracellular signaling pathways. The upregulation of SREBP-1 activates the cell cycle proteins and cell membrane biosynthesis, regulating cell proliferation [13]. SREBP-1 is overexpressed in cancer cells and is positively correlated with NF-kB activation [29]. It also promotes lipid desaturation through fatty acid desaturase and induces NF-kB signaling to stimulate RCC cell proliferation [30]. Furthermore, the up-regulation of SREBP-1 has been shown to be associated with malignant transformation and drug resistance mechanisms in various types of cancer cells [25,31]. This study showed that RCC 786-O cells stimulated by visfatin induced SREBP-1 expression and attenuated the cytotoxicity of sunitinib on RCC cells, while inhibition of visfatin-induced SREBP-1 expression could enhance the effect of sunitinib on 786-O cells. Experimental analysis also found that SREBP-1 upregulation was mediated through intracellular activation of Akt and p70S6K signaling. Phosphorylation of Akt and p70S6K has been shown to play important roles in regulating the chemoresistance mechanism of cancer cells through anti-apoptotic effects and promotion of cancer cell growth [32]. The Akt and mTOR signaling has also been reported to regulate SREBP-1 expression in numerous cell types [32,33]. Activation of Akt-mTOR signaling pathway has been reported to induce cancer cell resistance to ferroptosis through SREBP-1-mediated monounsaturated fatty acid production, whereas inhibition of this intracellular signaling can enhance the susceptibility of cancer cells to ferroptosis induction [34]. However, the visfatin-induced gene expression and signal transduction requires a mechanism-based approach to elucidate how SREBP-1 contributes to RCC cytotoxicity. Akt and p70S6K signaling pathways and downstream gene expression in cancer cells may be therapeutic targets for developing anticancer strategies in patients with obesity.

ROS has essential regulatory roles in a number of biological processes [35]. Under basal conditions, the critical function of ROS is to maintain cell proliferation and homeostasis. When excessive ROS production leads to an imbalance, it will induce oxidative stress and cause oxidative damage to intracellular molecules, thereby promoting the development of cancer [36]. Visfatin activates signaling pathways by increasing ROS, while ROS can activate Akt and p70S6K signaling pathways to upregulate the expression of many genes. However, we found that visfatin increased SREBP-1 expression through Akt and p70S6K pathways due to increased oxidative stress induced by visfatin.

Statins are specific inhibitors responsible for inhibiting de novo biosynthesis of cholesterol and non-sterol isoprenoids [37]. In particular, they inhibit HMG-CoA reductase, involved in converting HMG-CoA to mevalonate [37]. Previous studies have shown that the signaling pathway for the anticancer effect of simvastatin has been demonstrated to be related to Akt and p70S6K signaling activation and regulation [38,39]. Administration of simvastatin can enhance the cytotoxicity and efficacy of anticancer drugs on cancer cells [14]. Furthermore, statin suppressed the function of NF-κB in colorectal cancer cells [40] and enhanced the therapeutic effect of tyrosine kinase inhibitors in patients with non-small cell lung cancer [41]. In breast cancer cells, statin has also been found to enhance the anticancer effects of chemopreventive agents by inhibiting the mTOR pathway [42]. Treatment strategies of statin combined with other anticancer drugs may be more effective than monotherapy. In this study, the inhibition of SREBP-1 expression by simvastatin enhanced the sunitinib-induced cytotoxicity in visfatin-treated 786-O cells. Further, the possible mechanism by which statin and sunitinib combination treatment induces RCC cell death has also been explored.

## 5. Conclusions

Our study demonstrates that simvastatin adds a cytotoxic effect with sunitinib to RCC 786-O cells by suppressing visfatin-induced SREBP-1 upregulation. Although this study found that inhibition of SREBP-1 may enhance the therapeutic effect of sunitinib in RCC patients, the role of statins, visfatin, and their combination therapy strategies in obesity situations in vivo still needs to be further studied. It is worth noting that the combination strategy of statin and sunitinib can be used as an important concept for treating RCC chemoresistance.

## Data Availability

The data that support the findings of current study are available from the corresponding author upon reasonable request.

## Supplementary Materials

The following supporting information can be downloaded at: https://www.mdpi.com/article/10.3390/life12111890/s1, Figure S1: Original, representative blots corresponding to Figures in this study.
