# Supplementary material for: Suppression of SREBP-1 Expression by Simvastatin Decreases Visfatin-Induced Chemoresistance to Sunitinib in Human Renal Carcinoma 786-O Cells
Source: Life (Basel). 2022 Nov 15;12(11):1890. doi: 10.3390/life12111890 (PMC9695258; doi:10.3390/life12111890)

Figure S1. Original, representative blots corresponding to Figures in this study.

Figure 1C

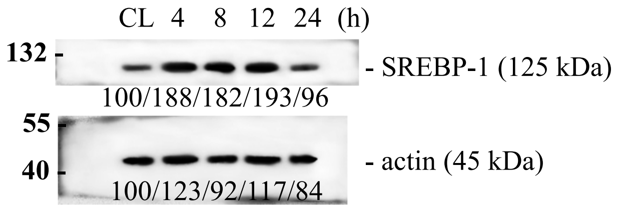

Figure 1D

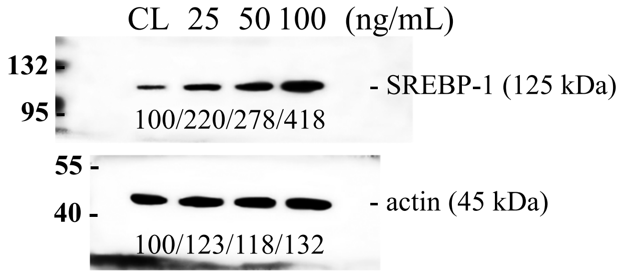

Figure 4C

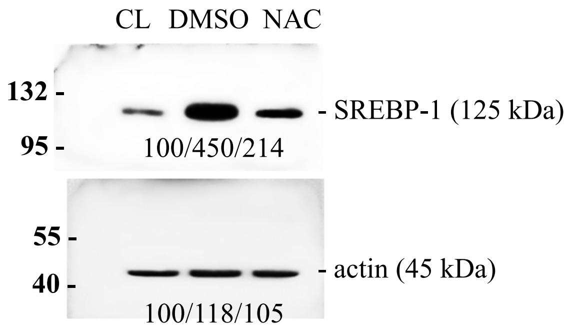

Figure 5C

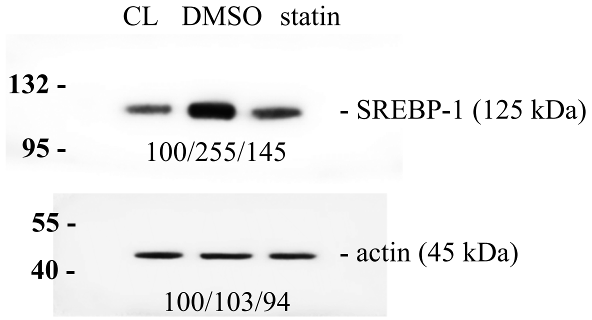

Figure 3B

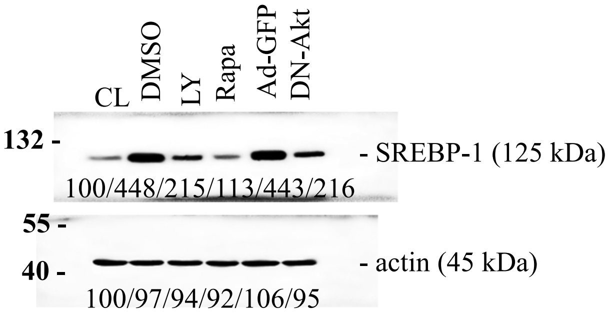

Figure 3D

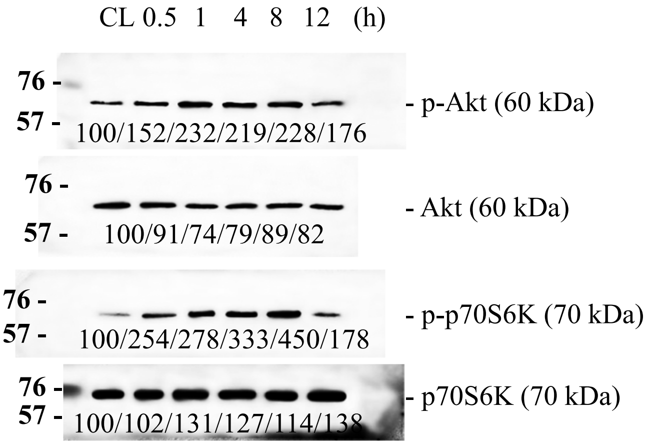

Figure 5D

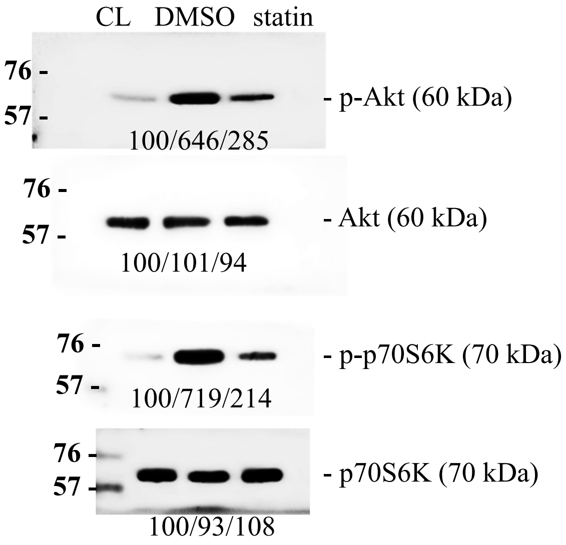

Supplement: Supplementary file 1 [file life-12-01890-s001.zip › life-1898449-supplementary.pdf]
